# Evaluating an interactive tool that reasons about quality of life to support life planning by older people

**DOI:** 10.1177/20552076241255633

**Published:** 2024-06-07

**Authors:** Neil Maiden, Sophie Hide, James Lockerbie, Simone Stumpf, Juanita Hoe, Shashi Hirani

**Affiliations:** 1 4895 Bayes Business School, City, University of London, London, UK; 2 3526 School of Computing Science, University of Glasgow, Glasgow, UK; 3 7364 School of Medicine and Biosciences, University of West London, London, UK; 4 School of Health Sciences, City, University of London, London, UK

**Keywords:** Older people, dementia, Parkinson's disease, quality of life, life planning, summative evaluation

## Abstract

**Objectives:**

In response to the lack of digital support for older people to plan their lives for quality of life, research was undertaken to co-design and then evaluate a new digital tool that combined interactive guidance for life planning with a computerised model of quality of life.

**Method:**

First, a workshop-based process for co-designing the SCAMPI tool with older people is reported. A first version of this tool was then evaluated over eight consecutive weeks by nine older people living in their own homes. Four of these people were living with Parkinson's disease, one with early-stage dementia, and four without any diagnosed chronic condition. Regular semi-structured interviews were undertaken with each individual older person and, where wanted, their life partner. A more in-depth exit interview was conducted at the end of the period of tool use. Themes arising from analyses of content from these interviews were combined with first-hand data collected from the tool's use to develop a description of how each older person used the tool over the 8 weeks.

**Results:**

The findings provided the first evidence that the co-designed tool, and in particular the computerised model, could offer some value to older people. Although some struggled to use the tool as it was designed, which led to limited uptake of the tool's suggestions, the older people reported factoring these suggestions into their longer-term planning, as health and/or circumstances might change.

**Conclusions:**

The article contributes to the evolving discussion about how to deploy such digital technologies to support quality of life more effectively.

## Introduction

The importance of healthier ageing and the quality of life of older people is now well established. For example, the World Health Organisation (WHO) recognised this importance a decade ago in its quest to ‘add life to years’,^1^ and policies that promote healthy ageing to support older people to remain active, valued and engaged citizens for as long as possible are advocated by governing bodies such as the European Commission.^2^ To implement these policies, practical guidance for informal carers is now readily available.^3^ In parallel, an increasing number of research studies have explored what healthy ageing and quality of life mean to older people.^4^

However, older people living at home, especially with chronic illnesses such as dementia and Parkinson's disease, can still face considerable challenges remaining healthy and maintaining quality of life,^5^ especially if living alone without sufficient support.^6^ These challenges may take many forms. For example, both falls and the fear of falling can impact negatively their quality of life.^7^ Reduced physical activity can lead to poor nutrition^8^ and there can be negative emotional impacts from a reduced social network and subsequent growing isolation^9^ and loneliness.^10^

Enabling the adoption of constructive behaviour among older people can facilitate their activities and social connections. The COM-B model of behaviour^11^ is now widely used to identify what needs to change for a behaviour intervention to be effective. Three key factors need to be present: capability, opportunity and motivation. However, often, the opportunity resources needed to change behaviour are not available to everyone when needed. Public funding for healthier ageing competes with other demands such as education and defence, and the proliferating number of private services for life planning^12,13^ are not accessible and/or affordable by all. Alternatives, such as online directories of possible activities to undertake,^14,15^ offer only general support and are not linked explicitly to personalised quality-of-life needs. The research reported in this article sought to fill this gap and to deploy a new interactive digital tool to support individuals living at home to explore tailored activities that support them to remain healthy and maintain quality of life.

The new tool, called SCAMPI (Self-Care Advice, Monitoring, Planning and Intervention), combined interactive guidance for life planning with a comprehensive and computerised model of quality of life. It was designed to support older people living at home to set up and maintain a life plan with which they could prioritise different quality-of-life goals and explore automated suggestions for different meaningful activities that the model associated with the prioritised goals.

The research followed a design science approach – one that sought to develop and investigate artefacts that interact in and with a problem context, to improve something in that context.^16^ Following this approach, the authors researched and co-designed a new artefact – the SCAMPI tool – that they analysed in the context of older people living at home, to investigate whether it had the potential to improve these peoples’ qualities of life. The multi-stage development of the computerised quality-of-life model was reported previously in a study by Lockerbie and Maiden.^17^ This article summarises the tool's co-design with older people living with different chronic conditions. It then reports an in-the-wild evaluation of the tool with nine older people living in their own homes, some of whom were living with the early stages of Parkinson's disease or dementia. Each was requested to use the tool regularly to record, investigate and make decisions about suggested meaningful activities associated with their prioritised qualities of life goals, over a continuous period of 8 weeks. Data was collected from the people and tool regularly during the evaluation and after it to answer research questions related to the tool's use, whether this use led to changes in both their planned and actual activities, and whether there was any perceived impact on quality of life.

The remainder of this article is in four sections. After reporting selected related work, the article describes the co-design of the SCAMPI tool. It then reports the evaluation method and results from tool use by nine older people including four living with Parkinson's disease and one with dementia. It ends with a discussion about the results and their validity, conclusions and next steps to develop and exploit elements of the SCAMPI tool.

### Related work

The WHO defines quality of life as not only the absence of disease or infirmity but also the presence of physical, mental and social wellbeing.^1^ However, few reported models of quality of life have the breadth of this definition. Disciplines, for example, health and nutrition, have developed models that, although informative, describe quality of life from one or more viewpoints.^18,19^ Alternative models were sought to design the planned SCAMPI tool.

#### Models of quality of life and behaviour change

One model recognised as the most pervasive influence on conceptualising quality of life in older people^20^ was reported by Lawton.^21^ Lawton's original research sought to extend gerontology to understand how older people with dementia experienced quality of life. It has provided a baseline for many care practices and frameworks such as the dementia quality-of-life instrument^22^ and Bath Assessment of Subjective Quality of Life in Dementia.^23^ The Lawton model defined six domains of quality of life: the *ability to perform activities of daily living (ADLs)*, *engaging in the meaningful use of time*, *competent cognitive functioning*, *physical health*, *socially appropriate behaviour*, and a *favourable balance between positive and negative emotion.*^21^ These domains aligned with the WHO's recognition of the need for physical, mental and social wellbeing for all people, including older ones and not just those living with dementia or Parkinson's disease. Each provided an input to the design of a tool capable of guiding older people to plan for and experience more quality of life.

Other frameworks have been developed to help older people understand and communicate their needs and preferences for quality of life as a whole. One^24^ supported people to define their desired personal outcomes, and the framework reported in the study by Palacios-Ceña et al.^25^ was used to document a person's preferred meaningful activities. Both concepts – personal outcomes and meaningful activities – also provided inputs to tool's design to guide older people to plan for and experience more quality of life.

Changing behaviour is one intended outcome of life planning. Different theory-based strategies and models of behaviour change have been reported.^26^ One model of behaviour change is COM-B.^11^ This model identified three factors needed for behaviour to occur: the capability of a person to make the behaviour possible, attributes of that behaviour that make the change possible, and the person's motivation to achieve the behaviour. Motivation is based on feelings of want or need – attractions to anticipated pleasure or satisfaction, and anticipated relief from discomfort – both mental or physical, and both are required at moments of behaviour change.^27^ The claim is that successful behaviour change can benefit from planning for better quality of life – planning that necessitates discovering goals corresponding to these wants and needs, then scheduling behaviours – activities – that the person has the capacity and other attributes to undertake. These benefits of life planning informed the design of the tool to guide older people to plan for and then experience more quality of life.

#### Quality-of-life planning tools

Planning and preparing for later life have been associated with increased wellbeing in older age.^28^ Different life planning solutions have emerged, from simple paper-based templates such as *This is me*^29^ to more sophisticated non-digital methods^28^ and personalised planning services.^12,13^ In addition, a growing number of digital care planning tools that focus on peoples’ daily medical and care needs are also used by care services.^30,31^ However, most of these solutions lack the sophistication of the reported quality-of-life frameworks, and many are only available to older people able to afford the costs of private provision.

Digital technologies are one obvious means of delivering more affordable quality-of-life planning to people. Some studies have revealed an association between effective telecare and improved quality of life in older people,^32^ especially for people with better social welfare status and health conditions.^33^ A cluster-randomised trial of telecare use by older people revealed that it was unlikely to transform their lives.^34^ Multiple barriers continue to impede older adults with cognitive decline from using digital tools.^35^ However, none of these studies explored how digital tools for life planning impacted quality of life.

#### Digital tools to deliver care for other people

More broadly, interactive digital technologies that support older people in their lives have also been reported. The use of digital tools by people living with long-term conditions is increasing.^36^ Many were developed to support people living with dementia, for example,^37^ reported early work that utilised interactive multimedia to stimulate long-term memory as part of reminiscence therapy. Assistive technologies can make a significant difference in the lives of people with dementia if delivered at home in thoughtful and sensitive ways.^38^ The use of computing devices designed as furniture pieces by older residents to provide notions of home, intimacy and possessions with which to develop a sense of personhood.^39^ Older people made personal digital timelines using technologies designed to support the building of memory.^40^ A tool that allowed individuals living with dementia to share their artwork.^41^ Immersive interactions with virtual environments of familiar places and activities have improved some aspects of the physical and emotional wellbeing of people with dementia.^42,43^ People living with mild to moderate dementia used existing technological and social resources to self-manage their lives.^44^ And a large-scale trial demonstrated how assistive technologies and telecare can support more independent living by older people with dementia.^45^ However, although some of these tools were demonstrated to contribute to some aspects of quality of life, none supported planning for it, or even made reference to the existing and established quality-of-life frameworks referenced in care practices. Indeed, no tools that digitise the application of these frameworks to support quality-of-life planning appeared to have been reported at the time of the study.

Fewer digital technologies have been developed to support older people living with other chronic conditions such as Parkinson's disease. Technology-enhanced care for people with the disease has had limited impact.^46,47^ Digital health projects have not lived up to the expectations of people living with Parkinson's disease,^48^ and although digital therapeutics with the potential to provide personalised interventions to manage Parkinson's disease, the techniques have remained underdeveloped.^49^ A more recent co-design of a digital companion for self-care revealed that people living with Parkinson's disease needed personalised support for their daily living.^50^ Again, none of these digital technologies available at the time of the study supported quality-of-life planning for older people living with Parkinson's disease.

### The SCAMPI tool

The SCAMPI tool was an interactive web browser application optimised to operate on tablet and devices and support older users living in their own homes to develop and maintain a life plan. Each life plan was a digital artefact that outlined the older person's goals and desired outcomes, life preferences, constraints, and activities to undertake to achieve their goals and outcomes. The tool suggested meaningful activities to users to achieve prioritised life goals. Its functionality and user interface were co-designed using workshops and empathy probes with older people living with the early stages of dementia and Parkinson's disease, then refined by walking through prototypes. These older people also worked with their carers to feedback on the tool's goal modelling. The tool had two major components – the computerised quality-of-life model and the interactive application.

#### Designing user interfaces with the end-users

Departing from previous co-design work with vulnerable users,^51–53^ we applied a new persona-centred approach called PERCEPT (PERrsona-CEntred Participatory Technology).^54^ At the heart of PERCEPT was co-creating personas with target users to shape the design of user interfaces.^55^ The process consisted of four workshops, each lasting about 3 hours, spaced about 6 weeks apart over 6 months, involving a group of five people living with Parkinson's and a group of two people with dementia and their informal carers. We decided to separate the people with Parkinson's and people with dementia and their informal carers, to keep workshops to a reasonable size and manage their different needs. Initially, three older people with dementia were recruited, each of whom had an informal carer.

In workshop 1 we focused on exploring the users. Each gave participants the opportunity to talk about their background, hobbies, interests, the technologies they interacted with, and the activities and goals they do or would like to achieve using structured tasks within a workshop setting. We co-created five personas during these workshops.^55^ The people with dementia and their informal carers developed three personas, and the people living with Parkinson's two. We built on these personas in the subsequent workshops.

After these workshops, all participants were asked to self-report their daily activities, interests, challenges and difficulties using empathy probes.^56^ Participant photos and notes were used to enhance the personas for use in the second workshop.

In workshop 2, we collected feedback on the tool's computerised quality-of-life model and sensor technologies considered to support activity detection. The workshop had three parts. In the first part, the data collected during the first workshop and the empathy probes were used to reflect on the personas created. Based on this reflection, minor changes were made to the personas in the Parkinson's group. In the second part, we explored the model's goals and activities from user perspectives. Feedback on the mappings from activities to goals and their labels was taken forward into the tool design. The final part of the workshop explored how sensor technologies might be used at home using demonstrations of different sensor devices. The results were used to add use cases for monitoring activities to the personas.

In workshop 3 we co-designed the user interface using low-fidelity prototyping with post-its, pens and different user interface elements such as buttons, checkboxes and dropdown lists. The co-created personas’ activities (e.g. shopping and playing golf) and goals (e.g. to maintain personal interests and achieve an active mind) were used to encourage participants to create designs for a wider user base. Designs were developed for key tasks including setting up a life plan, exploring weekly achievements, and managing privacy. The resulting low-fidelity prototypes were simple but indicated important tool functionality to scaffold life planning and useful metaphors and familiar visual constructs such as a calendar for planning. After workshop 1, the researchers generated more refined prototypes based on the paper prototypes and earlier discussion.

Then, in workshop 4, we evaluated one prototype using an adapted cognitive walkthrough. We were inspired by the GenderMag method^57^ that combines the use of personas with a cognitive walkthrough to focus on gender differences in evaluating problem-solving software. Participants used the personas to step through a series of tasks and screens using an interactive prototype loaded onto a tablet computer. A researcher posed questions at each screen such as ‘will <the persona> see what to do next’ and ‘will <the persona> realize that he did the right thing’, following a simplified cognitive walkthrough method. Researchers took notes on participant responses to these questions using a form adapted from the kit available at http://gendermag.org/.

The walkthrough feedback was then integrated into the final refined prototype which was tested with further participants. The implementation of the tool's interactive application followed these final prototype designs.

#### Designing the computerised model of quality of life with domain experts

The computerised quality-of-life model provided automated suggestions about life goals and meaningful activities to contribute to these goals. Experienced professional carers provided feedback on early descriptive versions of the model. Each workshop presented a physical form of the model that the carers could feedback on, change and extend.^17^ A first descriptive version of the model was developed using five goal types for quality of life derived directly from the Lawton model^21^: *ability to perform ADLs maintained, emotional state (balance) maintained, physical health maximised, cognitive health maintained,* and *social life maintained.* Each type was then associated with personal outcome goal types important to older people. These goals were, by definition, specific to individuals,^24^ so a review identified numerous examples of personal outcome goals that were clustered to generate a smaller set of personal goal types. These types included: *communication skills maintained*, *perceived state of memory maximised* and *ability to concentrate maximised.* Different types of meaningful activities that people can undertake to improve their quality of life^25^ were then used to generate types of goals using these activities. Examples of these goal types included: *engaged with own home environment*; *engaged for continuity achieved* and *engaged in the company of others*. The personal goal types were then associated with the goal types derived from meaningful activities. All links between goal types defined different modalities, for example, whether achieving a goal of one type results in the achievement of another or merely contributes to it.

**Figure 1.**
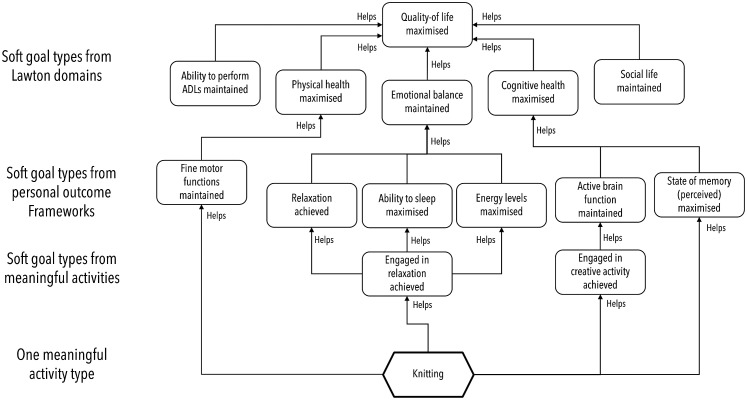
The five quality of life goal types used to structure SCAMPI's computerised quality-of-life model, and selected other goal types associated with them in the model, expressed using a simplified form of the i* language. SCAMPI: Self-Care Advice, Monitoring, Planning and Intervention.

The model was described using the graphical i* goal modelling language.^58^ It described 63 different goal types and over 200 typed links between the goal types. It was then tested with professional care staff to improve its accuracy and completeness.^17^ The model was extended to include an additional 744 meaningful activity types extracted from multiple sources including^59,60^ and over 30,000 typed links describing how completing each meaningful activity type contributed to achieving different goal types. Elements of one small part of this version of the model linked to just key meaningful activity type – knitting – are depicted visually using a simplified form of the i* language in Figure 1. The second, more complete version of the model was expressed using a machine-readable language with which to represent goals.^61^

The machine-readable version of the model was then implemented in a reasoning engine called CE-store.^61^ A total of 57 different automated queries were implemented to search over 50,000 model facts. The queries searched the model to discover types of meaningful activities that contributed to achieving quality-of-life goal types prioritised by a user and to compute the degree of progression against these goals based on completed activities of different types. The claim was that life planning using this computerised model of quality of life would trigger each older person to generate more goals in line with their capabilities, and from these goals schedule new activities that will lead to positive behaviour change.

#### The resulting interactive application

The co-design process revealed that many older people found life planning difficult, so the tool was designed to scaffold life planning. These people also wanted to use familiar visual constructs, so the tool provided an interactive calendar for this planning. Due to concerns about the reliability of internet access at home, most wanted the tool to be usable offline as well as online. Therefore, the tool was implemented using Hoodie^62^ that enabled offline use of web applications. It was optimised for use on tablet devices provided for the evaluation.

The tool was designed to provide interactive support for six key user tasks: (1) developing a profile including quality-of-life goal preferences; (2) setting up the life plan; (3) tracking suggested activities; (4) adding feedback to the calendar; (5) exploring weekly achievements and automated suggestions and (6) updating activities in the life plan.

##### Developing a profile

**Figure 2.**
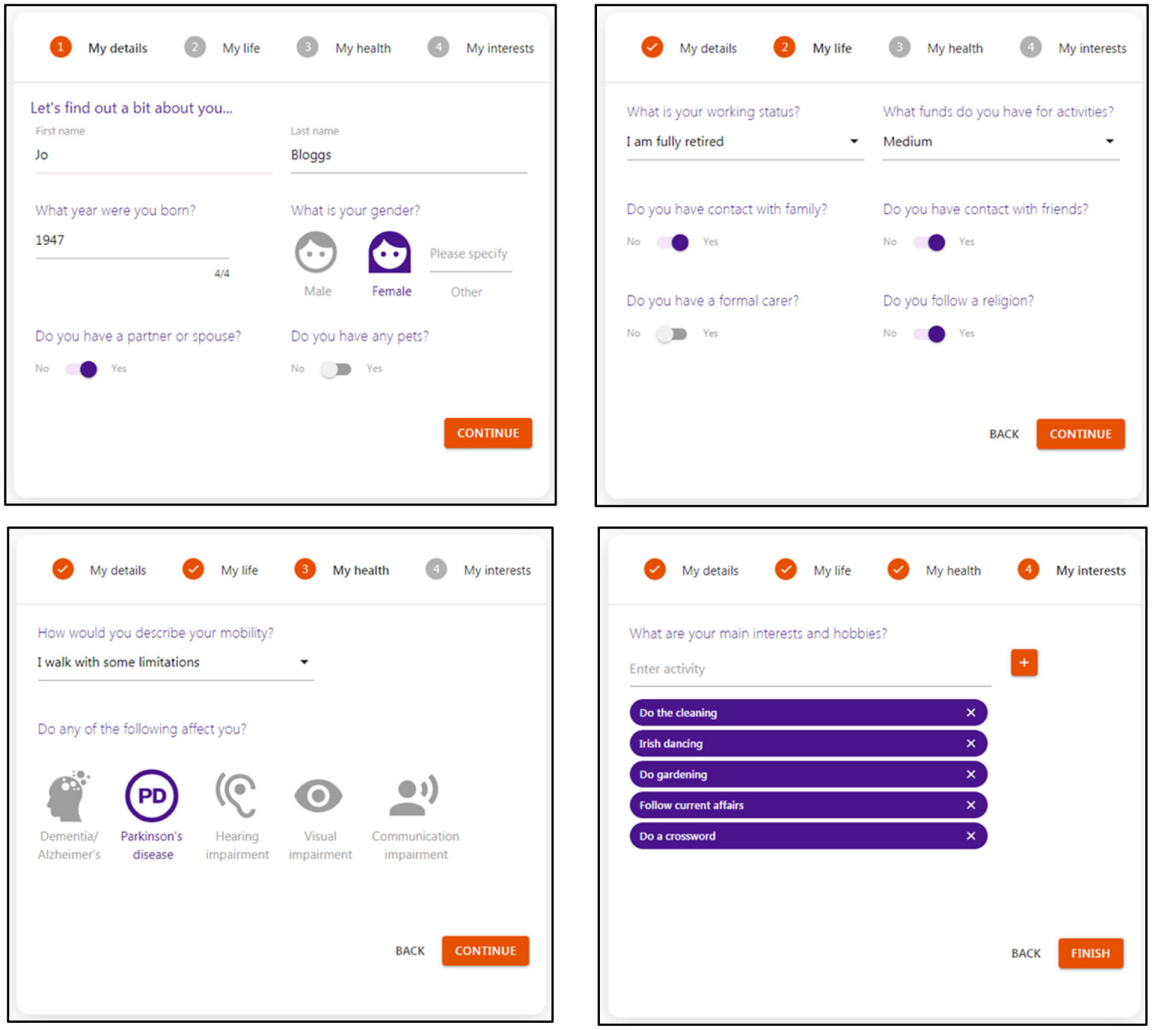
Examples of the SCAMPI tool's interactive features that enabled an older person to set up their profile in the tool. SCAMPI: Self-Care Advice, Monitoring, Planning and Intervention.

The tool walked a new user through steps to set up a personal profile by entering and selecting information about basic details, life, health and interests. This information included the user's gender, life details such as contacts with friends, health details such as mobility, and relaxation activities, and it was manipulated automatically by the computational quality-of-life model to generate tailored suggestions for meaningful activities, see Figure 2.

##### Setting up a life plan

**Figure 3.**
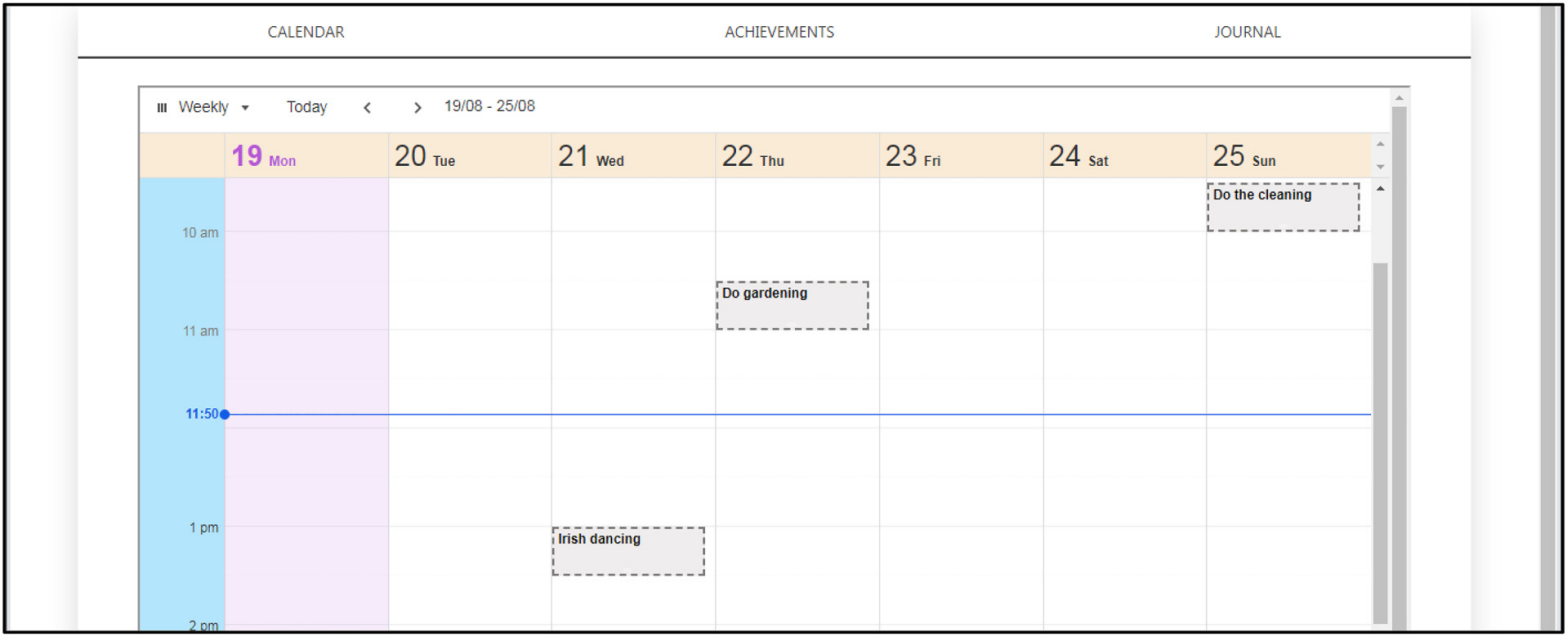
SCAMPI tool's visual life plan composed of meaningful activities that contribute to prioritised qualities of life, based on the design of online calendars. SCAMPI: Self-Care Advice, Monitoring, Planning and Intervention.

Each user's life plan was composed of a set of meaningful activities scheduled for specific days and times, each of which contributed to different quality-of-life goals as described in the computerised model. Each activity had a scheduled start and end time on one or more days of the week that enabled the user to plan routine activities such as eating and taking medication, see Figure 3.

**Figure 4.**
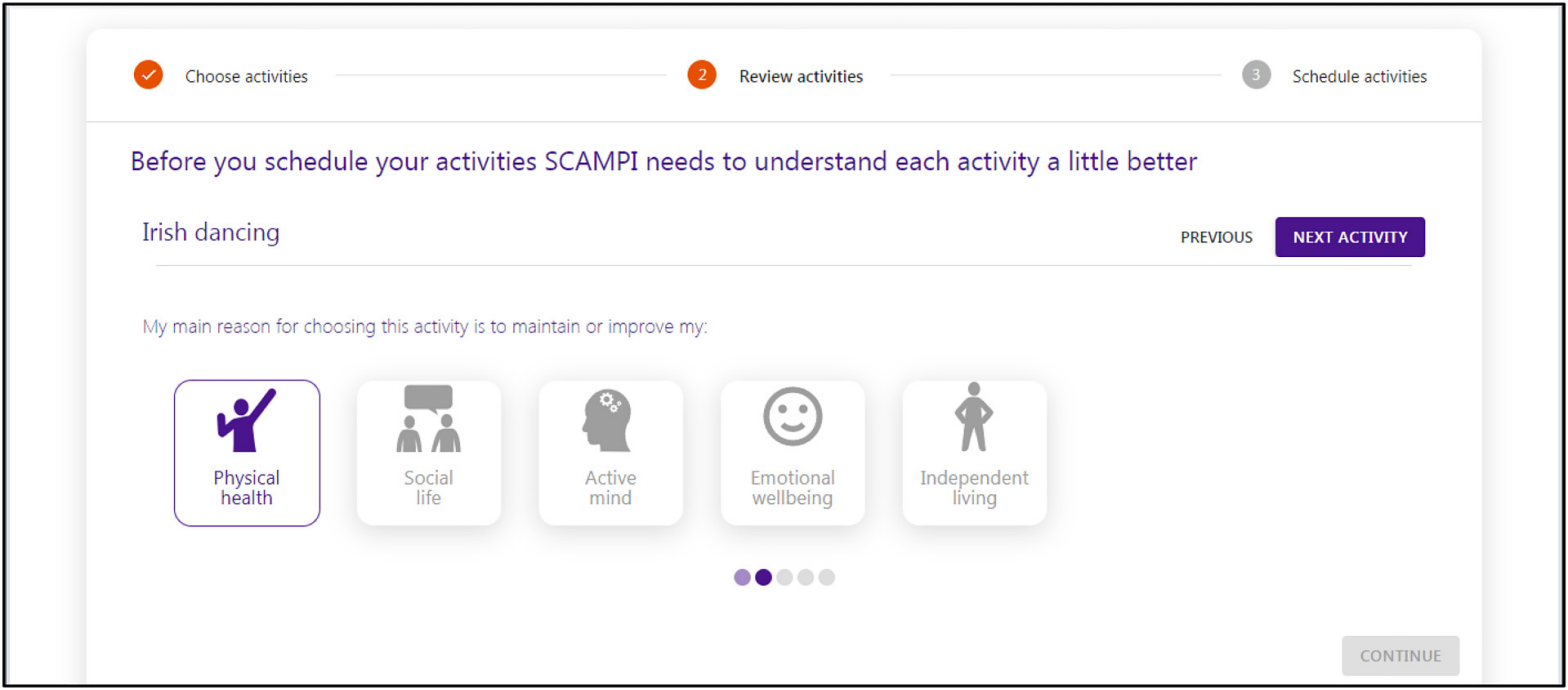
The SCAMPI tool's support for scaffolding a user's planned meaningful activities with selected types of quality of life, such as ‘physical health’ and ‘social life’, that correspond to soft goal types extracted from the Lawton domains. SCAMPI: Self-Care Advice, Monitoring, Planning and Intervention.

The tool also encouraged users to reflect on how each activity contributed to one or more quality-of-life goals, see Figure 4. Based on workshop feedback, it guided users to scaffold the plan by associating each activity with one or more of the five goal types derived from Lawton's model.^21^

##### Tracking suggested activities

During each week, users were able to select each scheduled activity and indicate whether it was completed, either fully or partially.

##### Exploring weekly suggestions and achievements

**Figure 5.**
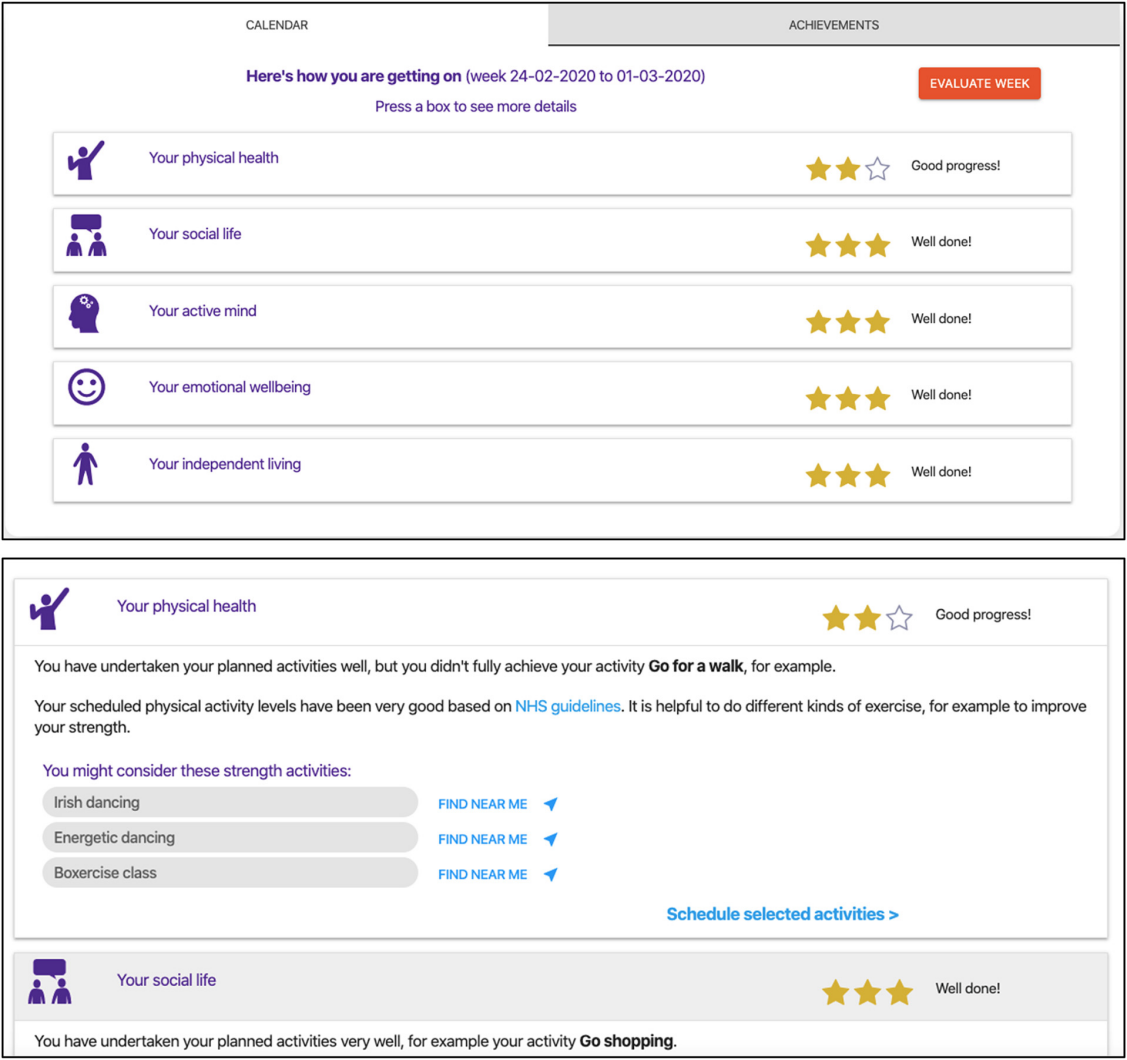
SCAMPI tool's interactive support for a user to explore their weekly achievements against soft goal types prioritised the life plan and new suggestions for activities. SCAMPI: Self-Care Advice, Monitoring, Planning and Intervention.

At the end of each week, the tool presented suggestions for new meaningful activities generated automatically by the computerised model's automated queries using the user profile information. It also presented their personal achievements in that week against the five quality-of-life goals from^21^ using simple star awards. The top of Figure 5 shows this user has completed most activities that contribute to four of the five qualities of life but fewer than contribute to physical health. Suggested activities generated by model queries shown in the lower screen to increase *physical health* include *Irish dancing* and *Boxercise class*.

##### Updating activities in the life plan

The tool guided each user both to maintain current activities and to incorporate suggested new ones into their life plans, as well as to add new activities of their own. Furthermore, it supported each user to explore activities in more detail, for example, to search for more information and local places to undertake activities.

## Method

Following the selected design science approach,^16^ the evaluation investigated the use of the SCAMPI tool by older people in their own homes, to understand possible and actual behaviour changes related to their quality of life. The evaluation took place between January and May 2020, a period affected by the first outbreak of COVID-19. Each person was living at home either with Parkinson's disease, early-stage dementia, or with neither condition. The evaluation sought to answer three research questions:

RQ-1: To what extent could these older people undertake the six tasks supported by the tool?

RQ-2: To what extent did the tool use support older people to document meaningful activities and quality-of-life goals before behaviour change?

RQ-3: To what extent did this tool support older people to change their activities and/or perceived quality of life?

### Approach

Each participant interacted primarily with one researcher experienced in both working with older people and in qualitative analyses with thematic techniques. The female researcher (the second author) was employed full time to undertake this evaluation work. She had a first career as a nurse and had also worked with older people (including those with dementia and Parkinson's disease) developing personalised care and support plans to enable them to sustain a range of daily living activities in their own homes. Her subsequent career incorporated both study (MSc, PhD) and education, research and practice in ergonomics. Each participant was made aware of the researcher's background during the first meeting, as well as her motivations for the research being undertaken.

During an introductory meeting, the researcher walked each participant through the purpose of the research and the evaluation procedure as it was documented in a participant information sheet distributed previously to all participants. She used these meetings to establish a personal relationship with each participant and, where needed, the participant's family carer, then obtained written informed consent following the research institution's required ethics procedures. The visits were personable in nature with a comfortable ambience and often involved a cup of tea. Each meeting was framed as a chat about topics ranging from where they lived, their personal histories and pets to their health conditions and the impact of these conditions on health and quality of life. As such, topics were introduced carefully into the conversation to ensure responses were elicited. Typical prompts were ‘*we’re really interested to hear a little more about your background, such as your health, work and milestones in life*’ and ‘*Would you be happy to tell me more about yourself?*’. Predefined questions on specific topics were avoided. There was a strong focus on each person's interests, to understand what motivated them. In response, if appropriate, the researcher shared some information about herself, to encourage more disclosure. Some participants shared their worries, for example, about their health or finances, and the researcher was able to use this information to contextualise questions during subsequent weekly interviews.

Following the introductory meeting, each participant completed a paper diary of their meaningful activities over 2 weeks. At the end of this period, each was given the chance to practice using the tool on a tablet device to set up their profile and life plan, then to plan and record meaningful activities over a continuous period of 8 weeks, during which the researcher sought to interview them weekly, based on their availability. Shortly after the end of this period, each engaged in a final exit interview with the researcher.

#### The participants and informal carers

Candidates were recruited from the earlier design workshops^55^ and networks such as the *Alzheimer's Society*, *Parkinson's UK* and other groups where older people congregate. If a candidate expressed an interest in participating, it was convenient for them to participate and their participation aligned with the aims of the research, an information sheet and consent form were sent by post and/or email at least 7 days before the first meeting. Vouchers were provided at the start of the study – £150 to a main participant and £45 to anybody offering a supporting role. Nine participants (6M, 3F) and three partners who acted as informal carers were recruited, see Table 1. Four had Parkinson's disease (PD), one had early-stage dementia (D), and four had neither condition (N).

**Table 1.** The identifiers for and basic information about the nine evaluation participants: PDx were participants diagnosed with Parkinson's disease, D1 was the participant diagnosed with dementia, and Nx were participants not diagnosed with either condition.

Code	Gender	Age	Self-report work status	Motivation to participate	Life partner in study?
PD1	M	67	Medically retired	Desire to support research, and living with Parkinson's disease	No
PD2	M	64	Self-employed	Desire to support research, and living with Parkinson's disease	Yes
PD3	M	54	Medically retired	Desire to support research, and living with Parkinson's disease	No
PD4	M	56	Employed	Desire to support research, and living with Parkinson's disease	Yes
D1	M	66	Medically retired	Desire to support research, and living with early-stage dementia	Yes
N1	F	51	Self-employed	Desire to support research, and prior association with people with dementia	No
N2	F	66	Retired	Seeking structure following bereavement, and desire to support research	No
N3	M	69	Retired	Seeking structure following bereavement, and concern about memory problems	No
N4	F	57	Housewife	Desire to support research, and prior association with people with dementia	No

*Note.* PD: Parkinson's disease; D: dementia; N: neither condition.

#### Preparation tasks

A first meeting to introduce and recruit each participant took place in their own homes. To demonstrate what tool use would entail, the researcher presented a short slide show and answered questions, then walked the participant and anyone with them through the information sheet. At no time was there pressure to divulge information that might be overly personal or upsetting.

**Figure 6.**
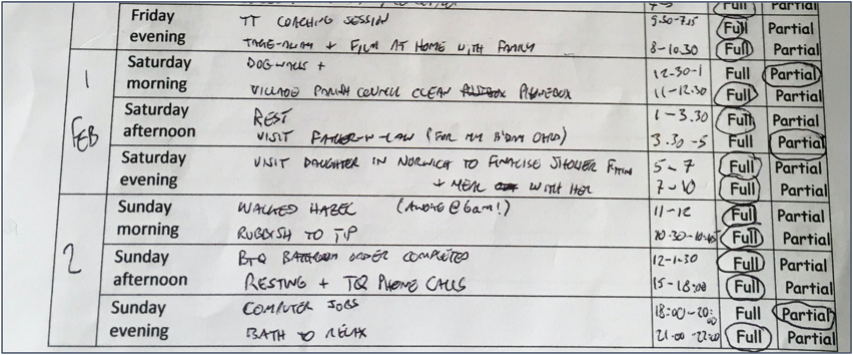
An example of a portion of one completed paper diary.

The researcher then sought to understand the participant's health, wellbeing, history and what was important to them. A simple paper diary was introduced, and each participant was invited to record daily activities for a 2-week period. It was explained that this would assist in building their life plan in the tool. To build up confidence with the diary, some participants were encouraged to make entries when the researcher was present, to receive reassurance that their entries were valuable and to uncover any issues that might have impeded participants from completing it. An example of a completed portion of one paper diary is depicted in Figure 6.

#### The tool and its handover

The tool and the tablet device that it ran on were introduced 2 weeks after the first meeting. The tablet was an unlocked Samsung Galaxy Tab A 10.1-in. 32GB with Wi-Fi but not SIM connectivity. Each participant completed their user profile with support from the researcher. Their life plan was compiled with details from the first meeting and paper diary – with the days of the week, times and durations specified by the participant. Some participants did this independently, others were assisted. Once compiled, the participant practised recording the extent to which planned activities were completed. All participants received a fully charged tablet, associated packaging and a charger. All were encouraged to contact the researcher (phone/email/text) if they had any questions or problems with tool use.

#### Regular tool usage tasks

During each week of tool use, each participant used the tool's features to indicate which planned activities were completed fully, partially, or not at all, to accept or reject each generated suggestion, and to update their life plan.

### Data collection via interviews

Semi-structured interviews with each participant in their own homes were planned for each of the first 6 weeks of tool use, and all apart from one with PD2 took place as scheduled. An exit interview with all participants also took place shortly after the end of the eighth week, apart from with D1, who was interviewed following the fourth week after he withdrew from the study. The life partners who were caring for PD2, PD4 and D1 were also invited to participate in and contribute to these interviews.

The first-week interviews were relatively flexible, which allowed both the researcher and participant to ask each other different questions. The researcher asked one set of questions based on the first interview topic guide in Appendix A and interventions needed to set up and use the tool. These topic questions were not communicated to the participants beforehand, but different forms of the same question were asked by the researcher each week, and this might have enabled some of the participants to predict the questions that the research asked. Each interview was conducted in a conversational manner, to develop rapport.

Subsequent interviews were undertaken both face to face in the participants' home and remotely using digital video calls with Skype or FaceTime. These interviews were more structured and asked questions about the participant's interaction experience, their achievements during the last week, and their responses to tool suggestions for new activities. These questions were based on the second topic guide in Appendix A. The researcher audio-recorded participant responses and took written notes that identified recording time points – this unobtrusive annotation enabled targeted searches of the audio records to capture such key points for data analysis. As participants became more proficient the discussions and data collected became more focused. Whereas the earlier interviews focused a little more on troubleshooting, the later ones asked and collected more information about both tool use and its potential impact on the participant's activities and life planning. Most of these weekly interviews lasted between 20 and 40 minutes but some were as short as 14 minutes and long as one hour in duration. For the final 2 weeks, each participant was asked to record their completion of planned activities without support from the researcher. This provided an opportunity to explore the extent to which each participant used the tool independently during each week.

The exit interview with each PD and N participant was held shortly after the end of the 8-week period of tool use. In contrast to the earlier interviews, it collected evidence of the overall impact of using the tool and reflection on life planning and quality of life. The researcher asked a series of questions about each participant's expectations of the tool at the start of the study, changes to their activities, behaviour and thinking as a result of using the tool, if and how tool use prompted other life changes, and reflections on the nature and importance of quality of life. The topic guide for these interviews is in Appendix B. The lengths of these exit interviews ranged from 39 minutes to 1 hour 30 minutes.

### Data collection using the tool

The tool automatically recorded different versions of each participant's life plan, including:

- planned activities and their duration;
- whether each activity was fully, partially, or not completed;
- uptake of any tool-generated suggestions;
- new activities not suggested by the tool, and;
- changes to documented quality-of-life goals.

The tool also recorded all auto-generated suggestions presented each week.

All of this data was collated from the tool, analysed at the end of the evaluation, and cross-referenced with data from the interviews.

### Protocol changes due to the COVID-19 lockdown

On 16 March 2020, the UK Government advised everyone in the UK against non-essential contact with others. The researchers pre-empted this advice and prepared two changes at the end of February 2020.

First, many meaningful activities incorporated into the tool's computerised model of quality of life took place outside and/or involved socialising, for example, *having a coffee with friends*, and *going to the cinema*. These suggestions were judged to be inappropriate during lockdown, so on 16 March 2020, the model was modified to disable 571 of the 827 activity types and add 15 new ones (e.g. *participate in online discussion groups*, *online exercise workouts*) more suited to social isolating. The revised model generated suggestions from a set of 271 meaningful types. The participants were at different weeks of the study – one was at week 7 whereas another was only at week 2, so most of their presented suggestions were generated from the modified model.

Second, the researcher only undertook all interviews remotely, via telephone, FaceTime and Skype. Participants were asked to email photos or screenshots of their completed calendar page for the week and the page summarising their achievements for that week before each interview.

### Data analysis

The weekly interviews were not transcribed fully due to a lack of time and our research focus on the exit interviews, nor were these transcripts returned to the participants for comment or correction. Instead, data analysis was based on the researcher's written notes as a form of journaling to capture thoughts with which to discover interview themes. Furthermore, the researcher used post hoc rationalisation in fortnightly research team discussions, to avoid missing nuances or issues that only later would be understood to be of importance, following.^63^

The information recorded during the interviews and research team meetings was structured using the six reported user tasks. Key timing points annotated during the interviews were transcribed and tabulated into three sections: (1) comments, questions and requests raised by participants, noting issues common among participants; (2) researcher observations on participants’ experiences including barriers and opportunities and (3) pertinent quotes.

By contrast, analysis of data from the exit interviews drew directly on Bryman's principles for thematic analysis^64^ based on Clarke and Braun’s study.^65^ This reflexive approach recognised possible collaborations in code development and acknowledged researcher skills to produce plausible and coherent data coding.

Therefore, all exit interviews were verbatim transcribed, then transcripts from four with different experiences of the tool (PD1, PD3, N2, N3) were printed for familiarisation. The researcher made hand-written notes of observations (early codes) in the margins of data insights, with another code to denote question responses that were related thematically. Once the first set of codes had been devised, all final interview transcripts were uploaded to sheets in MS Excel to complete the coding process. Interview question responses were annotated to indicate the participant, speaker, one main question code, and one sub-code. These main and sub-codes were developed in a separate document and reviewed iteratively, leading to some codes being amalgamated and others split. The full coding scheme is described in Appendix C. Where needed, new sub-codes were added to reflect comments. Further higher-level themes were then identified using latent analysis in response to the research questions about the tool's use, planned behaviour change, and perceived changes in each participant's activities, behaviour, outlook, or way of thinking as a result of tool use. This question generated a greater volume of information, so new coding schemes describing gained personal outcomes were applied. Finally, the themes were aggregated into key findings reported in the results.

Finally, quantitative data from the tool and qualitative data from weekly interviews were combined to reveal six categories of likely sources of planned activities: (1) already documented in paper diaries; (2) raised but already being undertaken by that participant; (3) proposed by the participant and not undertaken; (4) suggested by the tool but already undertaken; (5) suggested by the tool and not undertaken, and; (6) other. Examples of this coding process are not reported in the article, and each participant did not receive detailed outcomes from the evaluation beyond what relates to the quality of life and use of the tool.

## Results

Eight of the nine participants used the SCAMPI tool in each of the 8 weeks. The ninth, D1, who was living with early-stage dementia, stopped during the fifth week because of difficulties using the tool. Both he and PD1 struggled to press the tool's buttons successfully, and this was one reason for D1's withdrawal. These struggles continued even though the user interface design was changed to seek to avoid them. The team finally concluded that the problem was one common to older people using digital touchscreens. Older people can lose moisture from their skin and fingertips or experience abnormal thickening of the skin, both of which can also hinder electricity passing through to activate screens.

The weekly interviews revealed three other usability problems experienced by more than three or more of the participants:

- Wrong button presses resulting in unintended operations due to the close proximity of buttons, control arrows and keys on the pop-up keyboard, reported by D1, PD1 and PD3.
- Multiple unnecessary button presses, reported by N1, N2, N3 and PD3, although this was partially resolved by an early design change to ensure immediate visibility of a ‘tool busy’ circle.
- Navigation problems during operations involving multiple screens, reported by PD1, D1, N2 and N3. This was resolved by all users apart from D1 mastering the use of icons to retrace to the desired screen.

All of the participants accepted that the tool was a prototype, to be enhanced using their feedback. Eight of the nine used it to develop and maintain their life plans throughout the 8 weeks. The researcher observed that data saturation had not been achieved by the end of the 8 weeks.

### Life planning with the tool

The total numbers of activities scheduled in each participant's life plan by week are reported in Table 2. The median number of scheduled activities per week per participant was 12. The numbers reveal that each life plan was developed in one of two ways. PD1, PD3, N2 and N4 planned similar numbers of activities over the 8 weeks, whereas PD2, PD4, N1 and N3 started with simple plans, but at least doubled the number of planned activities in subsequent weeks.

**Table 2.** Total number of activities scheduled in each participant's life plan, by week.

Subject	Week 1	Week 2	Week 3	Week 4	Week 5	Week 6	Week 7	Week 8
PD1	9	8	9	8	10	9	7	8
PD3	14	16	16	16	18	17	11	10
N2	19	20	21	19	19	22	22	25
N4	9	9	12	14	13	14	9	9
D1	11	14	14	16	–	–	–	–
N1	4	21	27	24	21	21	15	26
PD2	3	3	5	6	10	11	11	12
PD4	6	10	12	13	16	18	22	21
N3	5	10	10	10	11	12	12	12

*Note.* PD: Parkinson's disease; D: dementia; N: neither condition.

The average total durations in minutes of the scheduled activities in each life plan per week across the 8 weeks are reported in Table 3. For each participant apart from PD4, these activities were scheduled to take on average between 2 hours 14 minutes and 6 hours 17 minutes per week. However, PD4's plan also included work activities, hence the higher total duration.

**Table 3.** The average total durations in minutes of the scheduled activities in each life plan per week across the 8 weeks (and 4 weeks for participant D1).

PD1	PD3	N2	N4	D1	N1	PD2	PD4	N3
247	145	190	222	174	134	416	3354	377

*Note.* PD: Parkinson's disease; D: dementia; N: neither condition.

The cohort of nine participants scheduled a total of 154 different types of activities in their life plans. Unsurprisingly given the individual focus of these life plans, only 16 of these 154 activity types were scheduled by three or more participants. These types are listed in Table 8. Of the 154 activity types, 115 were unique to one participant's life plan. Table 4 also reports the number of weeks that each participant scheduled each activity type.

**Table 4.** The total number of weeks that each participant scheduled a specific type of activity, for all activities scheduled to be undertaken by three or more different participants.

Activities	PD1	PD2	PD3	PD4	N1	N2	N3	N4	D1
Gardening	3		5	5		8		7	
Housework			8	8	6	8	8		
Listen to music	8		7		5	6			4
Take the bins out		4		7	5	1			3
Watch TV	4		5		1		8	8	4
Cook a meal		8			8			8	4
Grocery shopping			6	8	6			8	
Check health data	8		1			1			
Do the cleaning	8			3	4				
Family visit			5	2				6	
Go cycling			1	2	6				
Go shopping	8				3		8		
Go to work				7	2		4		
Stretching exercises			6	4	1				
Visit friend	2		6		3				
Walk the dog		8	8	6					

*Note.* PD: Parkinson's disease; D: dementia; N: neither condition.

The scheduled activities offered some insights into the preferences for activities according to each individual participant's age, gender, occupation, and condition. For example, the range of activities scheduled by D1 who was living with dementia was narrower than the activities scheduled by the other participants, and each participant living with Parkinson's disease scheduled some activities specific to the treatment of the condition. The four participants aged in their 50s initially scheduled more physical activities than the five participants aged in their 60s, but COVID-19 reduced these activities. By contrast, there was no evidence that the two female participants scheduled activities that were different from the seven males, or the two participants still employed scheduled activities different from those who were retired. Moreover, given the reports that risks such as falls can limit the quality of life of older people, there was no evidence that these risks restricted the activities that were scheduled.

Analysis of the quantitative tool data and weekly interview responses revealed that activities already being undertaken before the evaluation were the ones scheduled most frequently. These activities included not only social activities but also everyday activities such as laundry and shopping. Some but not all had been documented previously in the paper diaries. By contrast, few activities new to the participants, including activities suggested by the tool, were scheduled, see Table 5.

**Table 5.** Totals of activities scheduled in the eight participant's life plans, attributed to one of 6 source types reported in the weekly interviews.

	Identified by participant		Suggested by tool		
Baseline activities	Activity being undertaken	Activity new to participant	Activity being undertaken	Activity new to participant	Other
98	39	16	7	5	40

The weekly interviews revealed the participants’ reasons for rejecting the tool suggestions, see Table 6. The most common one was that the activities were being undertaken already but not in the plan. Another was the plan's rigid structure based on the design's metaphors, which impeded suggestions from being planned, or did not support planning irregular activities easily. Even when participants selected tool suggestions, the participants expressed caveats – for example, these schedules were hard to plan or for the future, see Table 6.

**Table 6.** Totals of different reasons reported more than once in weekly interviews for accepting and rejecting the tool's suggestions reported.

Decision to reject a suggestion	147	Decision to accept a suggestion	37
Because already doing the activity	39	New activity (but hard to plan)	11
Because already scheduled in life plan	30	New activity	8
Because activity too low-level to plan	12	New activity (doing, but not in plan)	7
Because activity cannot be planned	11	New activity, (but only in the future)	4
Because activity did not fit with life	7	New activity (and already in life plan)	3
Because activity did not occur regularly	6		
Because participant not interested in	5		
Because participant was unable to do	4		
Because participant lacked resources to do	2		
Because activity was not needed	2		
Because participant forgot about activity	2		

By contrast, few activities were rejected for reasons associated with quality of life, for example, they did not fit with their lives or was not interested to them. This was corroborated by the exit interview data, which revealed instead that the participants rejected suggestions for being inaccessible to them or age-inappropriate, see Table 7.

**Table 7.** Examples of reasons for rejecting tool suggestions reported in the exit interviews.

Feedback theme	Example exit interview quote
Inappropriate for current stage in life	The jog on the mini trampoline, probably not a good suggestion for somebody over my sort of age, who hasn’t done trampolining before [N2]
Inappropriate for time of year	Earlier this year, we were knee-deep in claggy muddy soil and I was getting prompts for going up to the allotment but frankly, you couldn’t go up to the allotment without waders [PD2]
Inaccessible	Then how does SCAMPI make a note to say this person cannot go out of the wheelchair. So, if I’m wheelchair-bound, I’m not going to go out – I don’t know how to put it. [PD1]
Lack of resources	But so then online exercise and online… and online treadmill session that you can get, which I didn’t even know existed. You have to have your own treadmill [PD4]
Effort to access	Possibly. I would – it's something that I would do as a day centre activity. But probably not something I’d do on my own [N2]
Poor fit to current preferences	I guess for me, like dusting, it just, you know I just thought that was just a silly suggestion. But I guess if you’ve got dementia, it's not a silly suggestion [N4]

*Note.* PD: Parkinson's disease; D: dementia; N: neither condition.

Overall, the results revealed that, even though eight of the participants maintained their life plans and reviewed the tool suggestions each week, these plans were rarely updated to include these suggestions. Instead, new activities added to the plans were identified by the participants themselves.

### Analyses of the interview data

Therefore, the coded data from the exit interviews were analysed to uncover further reasons for rarely updating the life plans to include suggestions, and to understand the potential behaviour and quality of life changes resulting from the tool's use. The topics that emerged from the analysis were aggregated into four major themes that are reported here: (1) benefits from the tool's use; (2) understanding of quality of life; (3) the value of scheduled activities and (4) using tool suggestions. Each theme is demonstrated with one or two pertinent quotes. Additional quotes supporting each topic are listed in Table 7. Minor themes that emerged from the analysis are not reported or discussed in this article.

#### Reported benefits of tool use

All but one of the participants reported benefits from using the tool, for example:

“SCAMPI has helped me to think about how I can improve my quality of life.” [N4]

Others reported being more active as a result of the planned routines, for example:

“Keeping a record of what I was doing… SCAMPI has made me it more positive for me…. Getting myself into a decent routine. Especially in keeping myself active.” [N3]

And at least four of the participants reported living more actively as a consequence of tool use. For example, one described a shift in mindset, to being more optimistic about what can be done, and empowering him in some form:

“So, there is a sense of – the frame of mind has changed a bit. Positive because it actually, sort of challenges me to think, yes, I can do it; yes, I can do this rather than can’t do this.” [PD2]

Multiple reasons for this were shared. One was the tool's suggestions, which were credited with creating new expectations and more tangential thinking, for example:

“It has changed my outlook and broadened… Again, because I suppose the suggestions coming up have sort of expanded it.”, and “It does trick you to think well yeah I should be doing more than just sitting watching the world go by outside.” [PD4]

At least four others reported a better balance of activities, for example:

“You just need this kind of nudge and a focus from time to time, dip in, and really focus on it and work out what you are doing to get back some kind – a more in-balance status” [PD3]

“So that sort of made me think about balancing my activities between – everything between intellectual, pleasurable and also just jobs that need to be done.” [N4]

Furthermore, rather than add new activities to their current plans, some participants reported planning for the longer term, for example, to plan as age and/or a condition progressed, even though the tool provided no explicit support for such planning, for example:

“I would say it might be useful but it's a matter of time, not right now, but it could be sometime in the near future.” [PD1]

The tool was also described as providing participants with greater ownership of their activities. It supported them to plan, rather than be responsive, and to be more reflective, for example:

“So, in a sense, I felt that I moved from someone who was observing my own life to someone who was participating in it and measuring its quality.” [N1]

However, many of the participants often reported the need for additional resources of different forms to undertake tool suggestions, for example:

“And my next one is affordability, what happens if I use my spare income.” [PD1]

Overall, the exit interviews revealed that use of the tool was valuable. Indeed, some participants claimed that, instead, the tool use provided greater ownership and control over their activities. It enabled them to gain personal insights by experiencing value through acknowledging small goals. However, the tool suggestions were treated more as stimuli for longer-term planning than for immediate change.

#### Understanding of quality of life

The tool's repeated presentation of the five quality-of-life domains from Lawton (1994) when exploring each week's suggestions and achievements, unpacked the meaning of quality of life for some participants, for example:

And it's certainly put a spotlight, or a little, a small spotlight, made me focus on it a little bit more and think of it more in practical terms. Rather than sort of, you know, quality of life fog out there that you vaguely think about.” [N4]

Furthermore, it increased participant awareness of these qualities when making decisions about rating the degree to which activities contributed to these qualities and selecting new ones, for example:

“But then again, it does make you think each time, actually well yes, well that hiking in a group isn’t only about the physical side. It's about the social side. So, it does make you think, ah, yeah actually – So that, yes. So yes, it built-in, that is good.” [N2]

“A mixture of—actually it's funny because it's a mixture of independent living, emotional wellbeing, and what was the social one? Social life, because the allotment is basically – there is quite a lot of folks I know, and I have known for quite a long time up there.” [PD2]

To conclude, this operationalisation of the qualities for simple decision-making tasks was reported to add value.

#### The value of planned activities

Participants also reported finding the week-by-week plan valuable. For example, one reported having been overwhelmed by everything they needed doing at once, but the tool prompted the participant to break the week down, for example:

“It's made me really think about what I do and what I look at … I thought I was reasonably structured … But having an actual schedule to work to is a really good idea for me.” [N2]

Three participants described the benefit that they experienced or promoted as beneficial for other future tool users in being supported or coached during tool use. This was seen as especially important for users less able to sustain motivation or to manage the process of seeking new activities alone, for example:

“I just feel, they’d struggle to either keep it up to date or lose that motivation and drive to do it on their own. So, it's got to be managed in some way.” [PD2]

“It needs this kind of dialogue to really test and challenge the individual. If they’re not motivated enough to do some of those things that should improve their quality of life, then I’m not sure if they would be motivated enough to actually use the tool on its own without some kind of commitment.” [PD3]

The COVID-19 lockdown also changed daily routines, and some participants reported positive benefits from tool use for life planning in this period of change, for example:

“So, this lockdown has been a good way to kind of- and it's certainly been quite interesting for me to work out my schedule. And how I’m going to fill my day to make it fulfilling and pleasant, and with tasks to do.” [N4]

However, participants also reported numerous problems when scheduling new activities due to the rigidity of the schedule's timings that did not reflect actual life practices, for example:

“I think in terms of my expectations were about time slots being only fifteen minutes, sometimes the kind of enjoyment and fulfilment of life happens in a moment and it's unexpected and can’t be prescribed for. So, inevitably, there would have been things that weren’t captured.” [N1]

As a consequence, life plans were reported to be incomplete, for example:

“So, the suggestions were, enter a competition, yeah, I send my crossword answers, usually to the Waitrose magazines, so that's something I would do. But I wouldn’t, at the moment, I wouldn’t put it on a diary. Look at photos on the computer. Again, that's something that happens automatically.” [N2]

In response, some participants requested more flexible planning features. Others modified their plans to avoid reporting activities that were not completed, and other reported some frustration with the tool's lack of transparency in its calculations about how achievements were computed, for example:

“I do a few little tweaks – it is quite easy to use – that's good – I take things out and put things in. I wouldn’t just take things out – everything out as otherwise you’d never have any reds.” [PD4] (‘Reds’ indicated scheduled activities not completed at the end of the week)

Overall, the digital life plans benefited some of the participants personally by structuring their time more effectively, even when the tool's implementation of this schedule was too rigid for many meaningful activities.

#### Using tool suggestions

Even though most tool suggestions were not incorporated into their life plans, over half of the participants reported that these were valuable and informed their decision-making, for example:

“As I say, I’ve been quite surprised how in-tune it seemed to be with the suggestion – I would say, perhaps eighty percent of the suggestions have been appropriate for me and things that could have appealed, even though I’ve not necessarily taken up on them now as I said, but something might have been added at some time in the future sort of thing. [PD3]

Moreover, the tool suggestions were recognised as valuable for other people, for example, relatives and informal carers:

“The application could be much more than just quality of life for the sufferer. But it could be really helpful for the, certainly a person who's helping somebody who has dementia. Or, a problem with being able to talk to other people” [N4]

The suggestions stimulated different ideas and directions, for example:

“No, I think it was something even like a couple of weeks ago, or two weeks ago it was, there was something, a really minor thing, but it said about an activity of playing indoor skittles or something. And I thought no, but I can get my putter out. [PD4]

“Having said that though, I found all the suggestions have made me think, even if they’ve not been appropriate. I think just having something to go on is better than a blank sheet of paper.” [PD3]

The suggestions sometimes challenged their expectations of what they considered what they were able to do. Furthermore, the process of considering these suggestions each week provided personal outcomes, although not all of the participants reported that the suggestions informed their decision-making.

Other quotes from the exit interviews that demonstrate the four major themes are listed in Table 8.

**Table 8.** Additional selected interview responses for each of the four topics

Topic	Interview responses
Reported benefits of tool use	“I think actually using the programme has been hugely beneficial.” [N2]
	“It has sparked off other threads and suggestions in my mind, even if what it came up with is not perfect, but it's been sufficient to trigger my own things – to do instead maybe.” [PD3]
	“I think I would actually substitute things … And if I fail to do something, or if I don’t think I’ve done enough, then I think, rather than thinking, oh well, that doesn’t matter. I think I would definitely substitute, look at other things that I would do.” [N2]
	“it's made me realise that this process is actually – the idea of it is making someone look, whether externally, or at myself, just to say what I do-do and what I don’t do.” [D1]
	“I tend not to make big shifts or changes in a hurry.” [PD2]
	“Yeah, I feel – has it changed my behaviour? No, I don’t feel – it has in a way because it's to reflect now … I view it more of a reflection than of an assessment of how I’m doing, not where do I want to get to.” [PD3]
Understanding quality of life	“So, if cycling helps me or any of the exercises help me then I will definitely do it. So, SCAMPI does definitely re-emphasise the importance of those five points.” [PD1]
	“But it's – why do you? Why is it good for you? But I’d never thought about it really” [PD4]
The value of planned activities	“And I think it will make me think about the friends that I’ve got, who are struggling … they’re definitely not fulfilling their physical goals. Because they haven’t got anybody to do things with. And I think that will be quite salutary … it will make me think more broadly about other people.” [N2]
	“If the weather was bad then I might, even just doing five minutes in between the rain would be sufficient than having to do more.” [PD3]
	“No, no. Because it's- they’re sort of tasks that, for me personally, I don’t schedule. You know, they’re just ongoing.” [N4]
	“Whereas in order to get it to work for me, I’ve had to – in terms of a more holistic, high-level view…. Yeah, it is more feedback… in terms of trends or progress.” [PD3]
	“I got 3 stars … what surprises me is that [the tool} gives an example of ‘Charity volunteer work’ [as a success], but last week just gone I deleted the amount of work on charity compared to my previous weeks (where I know I wouldn’t be doing some) so I did less amount of charity work last week compared to my previous week.” [PD1]
Using tool suggestions	“Things like that photography thing came up, and I thought, yeah, I’d be interested – that's something I think I would be interested in pursuing… And more of the exercise classes or online, probably a lot more online exercise I think I’d end up doing, as we go forward.” [PD4]
	“Yes, it sorts of sparks other ideas, even if I wasn’t going to do this, I could do something else that I hadn’t thought of previously” [PD2's partner]
	“Yeah, that is the kind of thing that could easily nudge me into that bigger project rather than just the personal activity part of it.” [PD3]
	“Dancing round the room’ … I hadn’t thought of doing that. That was definitely a, yes, going on the tip toes round the room. And actually, I think I’m … better on one foot than I could beforehand.” [N2]

*Note.* PD: Parkinson's disease; D: dementia; N: neither condition.

Finally, the exit interviews did not reveal any differences of note in the responses provided by the people living with dementia and Parkinson's disease and their family carers. One likely reason for this finding is the relatively few responses made during these interviews by these carers. Most family carer comments that were made supported those made by the participants.

## Discussion

This article reports the co-design and first evaluation of SCAMPI, a new digital tool that combined interactive guidance for life planning with a computerised model of quality of life that generated different meaningful activities to improve quality of life. Eight of the nine older people used the tool in each of the 8 weeks of the evaluation, and the results provided the first evidence that the tool, and in particular the computerised model, could offer some form of value to older people living both with and without chronic conditions. This support for people living with different conditions distinguished the tool from existing technologies developed to support specific aspects of the quality of life of older people living with, for example, Parkinson's disease,^49^ and dementia.^44^

Although the tool was used, it was not always used as designed, for example, most of the activities planned in the tool by the older people were already being undertaken before the evaluation. Many of the activities were also more fine-grained than anticipated. Five of the nine older people did not add many new activities to their life plans, and results revealed that this was because of the digital plan's rigid structure and usability limitations.

The results enabled us to answer the three research questions:

RQ-1: The older people undertook some but not all tasks supported by the tool successfully. Whereas all developed their user profiles, set up their life plans, added feedback to these plans, and explored weekly achievements and suggestions, most struggled to document new activities in the plans. Indeed, usability problems resulted in the older person with dementia withdrawing at the midpoint of the evaluation.

RQ-2: Perhaps as a consequence of this, most of the older people did not use the tool to document many new activities prior to changing their behaviour. However, they did report new expectations and ways of thinking about their future activities and balancing their current ones.

RQ-3: Furthermore, some of the older people reported changing their behaviour, in particular to live more actively during the evaluation period as a result of the tool's use. However, the tool's suggestions were rarely adopted during the evaluation period. Instead, the older people reported factoring these suggestions into their longer-term planning, as health and/or circumstances might change.

### Benefits from tool use

The latent interpretation and consideration of values reported in the exit interviews revealed that all but one older person benefited from the tool's use. Although rarely added to the life plans, tool suggestions were welcomed. For example, five of the participants scheduled and undertook gardening activities in their weekly plans. Of those who didn’t garden, one walked in the garden and watered the plants, and another visited public gardens. According to the computerised quality-of-life model, undertaking these activities regularly contributed to each of them achieving the modelled goal *engaged with nature*, which using the model potentially contributed to them *maintaining a sense of responsibility*, then *optimising their sense of self*, and ultimately *maintaining a balanced emotional state*. According to the model, undertaking these activities regularly also contributed to each of them *achieving engagement in physical activity*, which potentially could have contributed to each of them maintaining a healthy weight, and through that maximising physical health. Indeed, many of the older people reported living more actively, creating new expectations and ideas, and thinking in new ways about activities. Some also reported more ownership and control over these activities. In addition, the life plan's structure of weeks and days helped some but not all of the older people to schedule their lives more effectively. The visibility of different qualities of life was also positive and provided an awareness and understanding that was used in decision-making to select and balance activities.

However, unlike earlier studies that revealed an association between effective telecare and improved quality of life in older people,^32,33^ our evaluation revealed no evidence for improved quality of life associated with the tool's use during the evaluation period, a result also reported in the study by Gathercole et al.^45^ One reason for this might be the shorter period of tool intervention, a limitation that we return to in the section on limits arising from threats to results validity.

### Personalisation and flexibility

Although many of the older people reported that their life plans provided more structure to their lives, the specific design of the plan – slots of times on each day of the week appeared to be too rigid, for example, not all activities that the older people wanted to plan occurred regularly or had fixed start times or durations, and many were dependent on local factors such as weather. Instead, alternative and more flexible digital planning metaphors, such as to-do lists and daily suggestions tailored to local weather forecasts might have more potential.

Motivation was assumed to play an important role in the new activities, expectations and ways of thinking reported by the older people. West and Michie^27^ described motivation as being based on feelings of want or need that are either attractions to anticipated pleasure/satisfaction or anticipated relief from discomfort. Both were required for behaviour change. The results from our evaluation suggested that the older peoples’ feelings of want or need as defined in the West and Michie model were often longer term, beyond the 8-week duration of the evaluation.

Moreover, this focus on longer-term planning enabled some of the older people to negate their immediate lack of resources such as money, access to public transport, and people such as friends that impeded the immediate take up of activities. Motivation alone is insufficient for behaviour change to occur, and both a person's capability and attributes of that behaviour that make it possible are also needed.^11^ A redesign of the tool towards longer-term planning about activities can allow people to develop, with guidance, the capabilities and resources needed to undertake these activities. This redesign can also include richer user profiles and different types and levels of resources needed to undertake different types of meaningful activities.

### Increased sense of agency

Moore^66^ defined a sense of agency as a person's feeling of being in the driving seat when it comes to their actions. Older age has been associated with reductions in sense of agency due primarily to physical impairment^67^ that reduces the quality of life. In our evaluation, many of the older people reported an increased sense of agency arising from tool use that was manifest in deliberate decision-making about activities.

Different tool features might have contributed to this reported increase in sense of agency. Both the regular weekly process of sitting down to make deliberate yes/no decisions about suggested activities and the tool's concrete suggestions appear to have had some positive effects. Moreover, the externalised life plan both structured and provided greater ownership of these activities, and empowered the older people to control their lives more. However, the degree to which this sense of agency resulted in a change in quality of life was unclear, as priming thoughts about upcoming actions have been shown to foster an illusory sense of agency for those actions.^68^ Intention-behaviour gaps have revealed that intention can be a poor predictor of actual health behaviour change.^69^

Nonetheless, participating and measuring own life through the externalised life plan appeared to enhance self-awareness. This can be advantageous to the quality of life of older people. For example, lack of awareness has been described as a clinical feature of Alzheimer's disease,^70^ and self-awareness of falls risk in older people^71^ has been associated with rehabilitation engagement and motivation. Therefore, one possible design implication is to rethink the role of the digital life plan to promote greater self-awareness.

### The computerised model of quality of life

Overall, the computerised model was sufficient to provide meaningful feedback and guidance during each week of the evaluation. The breadth of the soft goal types in the computerised model from the definitions from World Health Organisation^72^ and Lawton^21^ definitions appeared to be sufficient to support decision-making about most meaningful activities. Likewise, the depth of the modelled soft goal types based on the personal outcome framework^25^ and existing taxonomies of meaningful activities^59,60^ appeared to be effective for suggesting valuable activities. Of course, the imposition of lockdown during the evaluation reduced the number of meaningful activity types to suggest by almost two-thirds, so further evaluations are needed post lockdown to determine the potential value of the original model. Nonetheless, the ease with which the model could be amended to account for contextual factors can be seen as a positive, and demonstrate amendments for different planning contexts, for example, wheelchair users and people with more advanced chronic diseases.

The development of the model also demonstrated the benefits of novel interdisciplinary work – in this case creative combinations of existing approaches from social care, computer science and design science. The research applied an established and scalable goal modelling language from software engineering^60^ to model goal types associated with quality of life, then transformed this model using the CE machine-readable language and related CE-Store commercial reasoning engine.^61^ The authors are unaware of any previous efforts to codify informal knowledge about older person care practices for automated reasoning about quality of life. Furthermore, our selection of the CE language, a subset of English that is both human-readable and machine-readable^61^ allowed the model of quality of life to be reviewed and validated by professionals and experts in dementia care.^17^

### Limits arising from threats to results validity

Of course, the results of the evaluation are subject to different multiple threats to their validity^73^ that limit the claims that can be made from them. The threats to construct validity limited the generality of results to the underpinning theories – in this case, the frameworks of quality of life from social care research and computerised goal modelling. The results demonstrated that computerising a goal model of quality of life from existing frameworks was possible. However multiple different models and implementations are available, and more research will be needed before we might claim that the model and its implementation can support older people in different contexts with different needs. Furthermore, this evaluation only explored the impact of reasoning with the computerised model's five quality of life domains and 271 meaningful activity types that could be explored legally during lockdown. As already stated, future post-pandemic evaluations are needed to explore the value of a more complete set of activity types derived from the reported personal outcome frameworks. As such, the current results are limited to an evaluation of activities that could be undertaken legally during one COVID-19 lockdown.

Threats to the validity of our conclusions about relations between the tool's introduction and the evaluation outcomes^73^ arose from other variables related to more active living and quality of life. During the evaluation, the participants were free to access sources of guidance to improve their quality of life. No other sources were reported in the exit interviews, but the use of these sources cannot be discounted. Clearly, the introduction of a lockdown during the evaluation changed participant behaviour and might have stimulated more active living, but again, the exit interviews revealed no direct evidence of this. Instead, participants attributed the behaviour change that was reported to tool use. Nonetheless, again, the current results are limited to an evaluation of the activities undertaken legally during the COVID-19 lockdown.

Threats to the internal validity of the evaluation were influences that could have affected independent variables related to causality.^73^ One obvious threat was that the participants modified their behaviour in response to the series of interviews, and these interviews did elicit some evidence that some participants were keen that use of the tool would impact their lives positively. The possible impacts of these semi-regular interviews limit the conclusions that can be drawn from the evaluation. Another threat was researcher guidance during these interviews that compensated for tool deficiencies. To overcome these current limitations, longer studies with a more diverse set of older people and reduced researcher engagement are needed. Finally, some exit interview responses revealed that some of the older people did not perceive the tool as one that they needed themselves, despite their own diagnoses, but instead participated in the evaluation on behalf of others who were more in need of support for their quality of life. Participation in the evaluation for the benefit of other older people might also provide another explanation for the lower-than-anticipated uptake of activities suggested by the tool. Finally, the different topic guides reported in Appendix A that informed each semi-structured interview were developed specifically for this evaluation by the researchers. As such, these guidelines had not been validated previously, and this created a risk and another limitation related to the accuracy and completeness of the collected data, with consequences for the strength of the conclusions that can be drawn from the qualitative evaluation data.

Finally, threats to the evaluation's external validity were conditions that limited our ability to generalise results from the evaluation of one tool with only nine older people. We do not present the SCAMPI tool as a go-to-market tool to enhance the quality of life. Instead, we merely position it as one demonstrator of how digital technologies can be applied to support quality of life in older people. Researchers are encouraged to evaluate it and other tools to explore similar research questions. Likewise, we do not present the evaluation results as definitive evidence that interacting with a computational model of quality of life can enhance life planning by older people. The evaluation period of 8 weeks revealed preferences to use the tool for longer-term life planning, indicating the existence of other themes that did not emerge during the evaluation. Instead, the evaluations on this topic are limited to one early pilot, albeit with a rigorous method. Readers should treat it as a pathfinder for other, future in-depth evaluations.

## Conclusions

In response to the lack of digital support for older people to plan their lives for quality of life, research was undertaken to co-design and then evaluate a new digital tool that combined interactive guidance for life planning with a computerised model of quality of life. The first version of the SCAMPI tool was evaluated over eight consecutive weeks by nine older people living in their own homes. Four of these people were living with Parkinson's disease, one with early-stage dementia, and four without any diagnosed chronic condition. Regular semi-structured interviews were undertaken with each individual older person and their life partner, and a more in-depth exit interview was conducted at the end of the period of tool use. The evaluation results provide some tentative evidence for decoupling the SCAMPI tool's interactive planning support from its model-based guidance and exploring how each can be developed further. For example, to increase the sense of agency through life planning, life planning support would be integrated into artefacts used more regularly by other people. These artefacts are not only digital calendars such as iCal and Outlook, but also household objects such as digital televisions, radios and kitchen appliances. Indeed, life planning devices designed as everyday objects such as wall calendars and furniture have been shown to enable a sense of personhood,^39^ and can also be used to enhance agency. Attributes of agency, such as empowerment, motivation and ownership, can be used to drive the design of these extended devices. More co-design workshops are needed.

The evaluation results pointing to the longer-term benefits of the computerised model of quality of life suggest more value as a decision support engine providing both general advice for older people and more fine-grained individual guidance. However, this individual guidance will need to be informed by richer user profiles that enable more tailored tool-generated feedback and suggestions. Therefore, we are currently exploring how future versions of the computerised model of quality of life can be integrated into services such as personalised planning services^12,13^ and commercial care services such as residential villages. We are also exploring how the tool can be integrated into digital care planning tools that focus on peoples’ daily medical and care needs^30,31^ for use by activity coordinators. Both integrations have the potential to extend these tools with more sophisticated quality-of-life guidance, perhaps to make them accessible and/or affordable by most older people.

Finally, the evaluation results revealed that some of the tool-generated suggestions for concrete meaningful activities were effective in stimulating effective creative thinking about new activities. The suggestions were often perceived to be too narrow and literal. Therefore, the next version of the computerised model of quality of life will also generate more generic suggestions intended to define and explore conceptual spaces of larger numbers of possible ideas for activities that older people are more likely to plan to undertake.

## Supplemental Material

sj-docx-1-dhj-10.1177_20552076241255633 - Supplemental material for Evaluating an interactive tool that reasons about quality of life to support life planning by older people Supplemental material, sj-docx-1-dhj-10.1177_20552076241255633 for Evaluating an interactive tool that reasons about quality of life to support life planning by older people by Neil Maiden, Sophie Hide, James Lockerbie, Simone Stumpf, Juanita Hoe and Shashi Hirani in DIGITAL HEALTH

## Appendix A: Topic guide for weekly semi-structured interviews

To collect data during the first week's semi-structured interview, the researcher guided the participants in set-up tasks and asked them questions. The key topics that informed these questions and guidance were:

- Completing their user profiles in the SCAMPI tool using the paper diaries, including ensuring their passwords worked, ensuring that the tablet device was useful and overcoming any issues with the SCAMPI user interface, helping with activity scheduling.
- Exploring usability and interaction issues during the walkthrough of the tool's use by each participant.

To collect data during the semi-structured interview each week, the researcher asked each participant about:

- Developing their user profile in the tool, a topic that became less relevant to ask about during the later weeks of data collection.
- Their use of the calendar, often by walking through it with them to collect data about each planned and achieved activity.
- Inputting feedback and exploring achieved meaningful activities, including questions about whether the activity might be continued or not, and whether it might be moved to other dates and times in the calendar.
- Controlling and navigating the tool, which was achieved by asking questions about ease-of-use, problems that had been reported and help sought, and observing any usability difficulties when the researcher was present.
- Investigating the achieved meaningful activities based on the tool's colour-coding of activities. Participants were also encouraged to provide reports of other activities undertaken that week that were not covered in the tool's life planning.
- The achievement or otherwise of different quality-of-life goals, based on the tool's auto-generated feedback to each participant. Most participants were curious to see these summaries and keen to elaborate on both successes, failures and their reasons that were not recorded in the tool.
- Reviewing and selecting between meaningful activities suggested by the tool. Participants were also curious and keen to review their suggested activities, and the interviews were effective at capturing reasons why activities might or might not be undertaken by them.
- Exploring more about each tool suggestion, and each participant was asked about the suggestion and the information accessible via the clicked hyperlink presented by the tool for each suggestion.
- Revising the individual life plan, and satisfaction with it. Each participant was asked about the plan as a whole, and about any specific changes including additions or deletions that the participant would have liked to make to the plan.
- Other feedback on the experiences and perceptions of the tool use and outputs, which acted as a catch-all topic to elicit other information not communicated with the above topics. This included a brief discussion of the conditions that each participant had reported, to understand their wider health and wellbeing.

## Appendix B: Topic guide for exit semi-structured interview

Each participant was asked to report:

- The impact of their experiences of using the tool.
- Whether, through the use of the tool, they had changed their activities, behaviour, outlook, or ways of thinking.
- How the transition to the revised ‘lockdown’ tools suggestions impacted them (Participant D1 was excluded from this question).
- Whether the use of the tool had encouraged them to reflect on the meaning of quality of life.
- Other unexpected benefits arising from tool use.

## Appendix C: Codified research themes from exit semi-structured interviews

A. Expectations (society/personal/practice)

- Tech targeted at the elderly/special health needs.
- Disappointment in the study – sensor trial or dull/stilted.
- Promotes QoL and safeguarding/SC domain.
- A chance to be part of something.
- Personal enjoyment.
- Retirement/ bereavement related.
- Plan ahead – day to day.
- More of a detailed diary.
- Follows co-design workshop aims.
- None.
- To have more local (home base) focus.

B. SCAMPI impact on activities, outlook, behaviour

- Value of having a plan/schedule to work to – gain focus; develop routine; self-motivation; prioritise/balance; review; achieve more.
- Acknowledging the importance of registering the small things/Value of small goals.
- Future activities – influenced by post-lockdown; suitable weather; adequate time; retirement.
- Distinguishing/meeting the QoL goals – achieving balance; acknowledging importance; curious about bigger picture; interpretation difficulties.
- Explore/challenge own expectations of what can be/was achieved.
- Self-determination – ownership; personal control of scheduling.
- Response to suggestions – worthwhile; prompt other ideas; good for others; value of unsuitable suggestions; they’re for older people.

C. Self-isolation related

- Alternative activity means adopted – change mindset.
- New ideas/lightbulb moments.
- Reminder/prompt – resurrecting old ideas.
- Difference not really noted/deemed a big influence.

D. Unexpected benefits/considerations

- Subsequent development of own diary/list.
- Coaching to 〈 motivation/colleague relationship with researcher.
- Consideration of others who might benefit/support other service providers.
- Better health/wellbeing.
- Own learning.

E. QoL meaning/impact

- Interpretation of info provided by SCAMPI – 〈active/independent living/achieve goals.
- Awareness of what can be lost – what the future might look like.
- Reflection on how time is used.
- None.

F. Outstanding wants/concerns

- Seeking ongoing features – self-supporting group/finalised SCAMPI product.
- Monthly summary of achievements + be able to review before/after SCAMPI use.
- For communication between different devices.
- Usability + simplification for target users + guidance for users.
- More appropriate suggestions.
- Clarify its diary/planner role and use as a forward or retrospective planner.
- Reason to use it – objectives or aims.
- Apply QoL goals to own diary.

G. Presentation

- Preference for a tablet.
- Preference for whatever folk already use.
- Standalone device versus upload to own tablet.
- Laptop/PC.
- Caution about a lack of App/IT experience/habit formation.
- Smartphone.

H. Funding

- Assoc./NHS.
- Private.
- Subscription.
- One-off.
- Variation on above.

I. Further feedback on SCAMPI features (not reported in final interview section – already addressed in main body of report)

- Concerning the suggestions and search.
- Concerning having a link across devices.
- Concerning usability and database related problems.
- Concerning fulfilment of goals/stars.
- About having a reminder.
- About paper diary not being as comprehensive as SCAMPI.
