# Supplementary figures and images for: Evaluating an interactive tool that reasons about quality of life to support life planning by older people
Source: Digit Health. 2024 Jun 7;10:20552076241255633. doi: 10.1177/20552076241255633 (PMC11159557; doi:10.1177/20552076241255633)

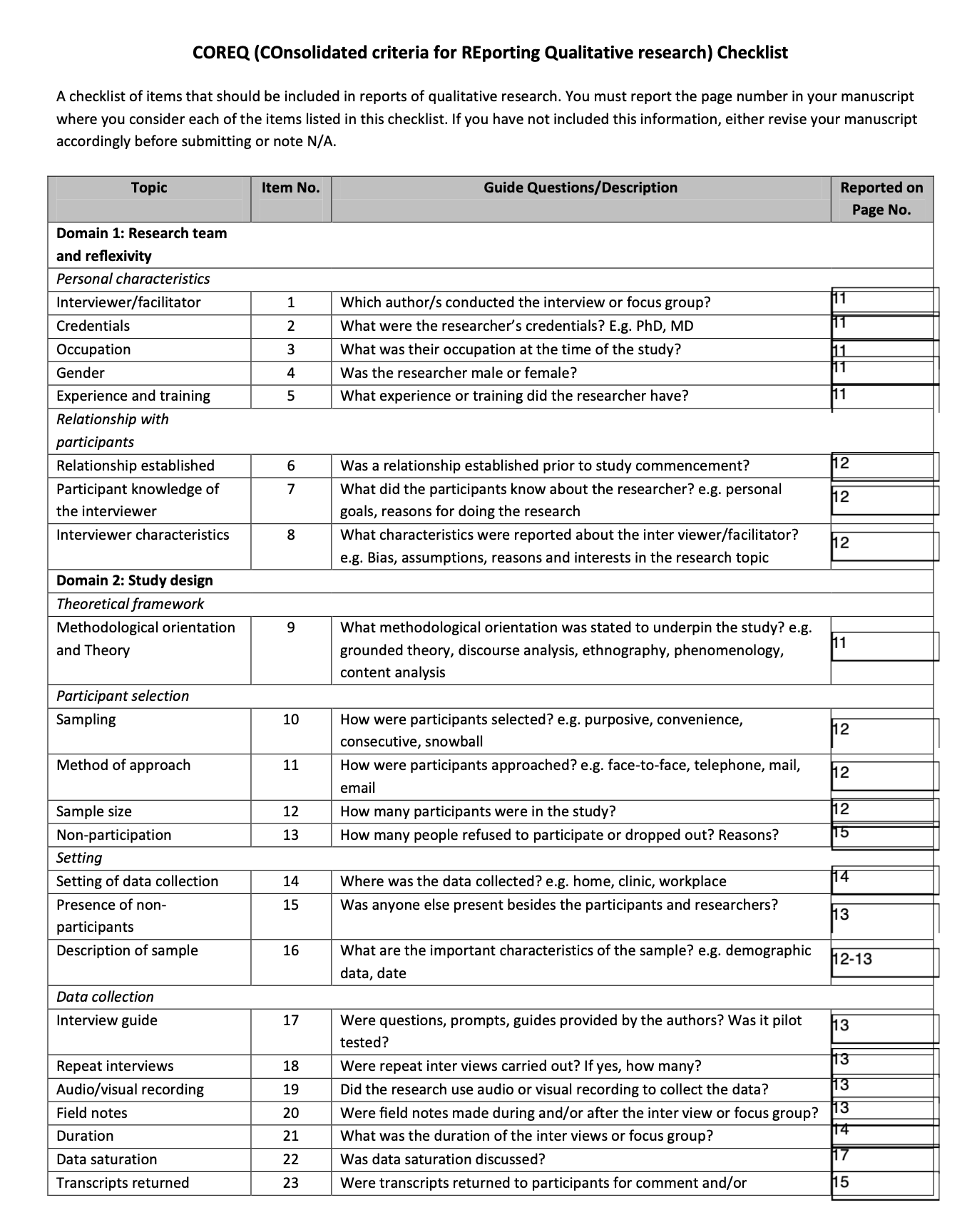


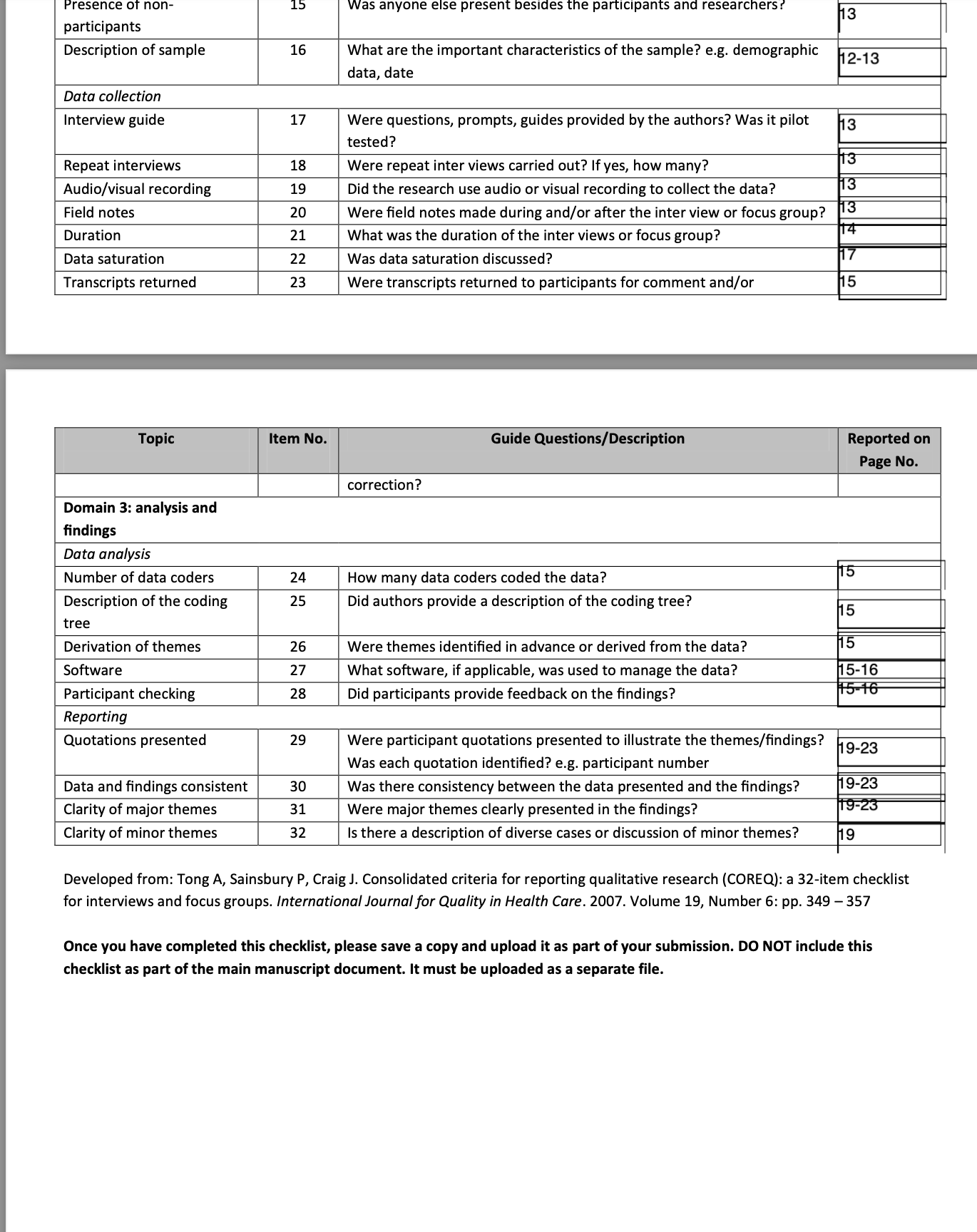

Supplement: sj-docx-1-dhj-10.1177_20552076241255633 - Supplemental material for Evaluating an interactive tool that reasons about quality of life to support life planning by older people [file sj-docx-1-dhj-10.1177_20552076241255633.docx]
